# EU-AIMS Longitudinal European Autism Project (LEAP): the autism twin cohort

**DOI:** 10.1186/s13229-018-0212-x

**Published:** 2018-04-13

**Authors:** Johan Isaksson, Kristiina Tammimies, Janina Neufeld, Élodie Cauvet, Karl Lundin, Jan K. Buitelaar, Eva Loth, Declan G. M. Murphy, Will Spooren, Sven Bölte, the EU-AIMS LEAP group, Jumana Ahmad, Sara Ambrosino, Bonnie Auyeung, Tobias Banaschewski, Simon Baron-Cohen, Sarah Baumeister, Christian Beckmann, Thomas Bourgeron, Carsten Bours, Michael Brammer, Daniel Brandeis, Claudia Brogna, Yvette de Bruijn, Bhismadev Chakrabarti, Tony Charman, Daisy Crawley, Ineke Cornelissen, Flavio Dell’ Acqua, Guillaume Dumas, Sarah Durston, Christine Ecker, Vincent Frouin, Pilar Garcés, David Goyard, Lindsay Ham, Hannah Hayward, Joerg Hipp, Rosemary J. Holt, Mark H. Johnson, Emily J. H. Jones, Prantik Kundu, Meng-Chuan Lai, Xavier Liogier D’ardhuy, Michael Lombardo, David J. Lythgoe, René Mandl, Luke Mason, Andreas Meyer-Lindenberg, Carolin Moessnang, Nico Mueller, Laurence O’Dwyer, Marianne Oldehinkel, Bob Oranje, Gahan Pandina, Antonio M. Persico, Barbara Ruggeri, Amber Ruigrok, Jessica Sabet, Roberto Sacco, Antonia San José Cáceres, Emily Simonoff, Julian Tillmann, Roberto Toro, Heike Tost, Jack Waldman, Steve C. R. Williams, Caroline Wooldridge, Marcel P. Zwiers

**Affiliations:** 1 Department of Women’s and Children’s Health, Division of Neuropsychiatry Unit, Center of Neurodevelopmental Disorders (KIND), Karolinska Institutet, Stockholm, Sweden; 2 Department of Neuroscience, Child and Adolescent Psychiatry Unit, Uppsala University, Uppsala, Sweden; 3 Child and Adolescent Psychiatry, Center for Psychiatry Research, Stockholm County Council, Stockholm, Sweden; 4 Department of Cognitive Neuroscience, Donders Institute for Brain, Cognition and Behaviour, Radboud University Medical Centre, Nijmegen, The Netherlands; 5 Sackler Institute for Translational Neurodevelopment, Institute of Psychiatry, Psychology and Neuroscience, King’s College, London, UK; 6 Department of Forensic and Neurodevelopmental Sciences, Institute of Psychiatry, Psychology and Neuroscience, King’s College, London, UK; 7 Roche Pharma Research and Early Development, Neuroscience, Ophthalmology and Rare Diseases, Roche Innovation Center Basel, Basel, Switzerland; 8 Child and Adolescent Psychiatry, Stockholm County Council, Stockholm, Sweden

**Keywords:** Europe, Autism spectrum disorder, ADHD, Twins, Genetics, Brain, Cognition, Biomarkers, Intervention

## Abstract

EU-AIMS is the largest European research program aiming to identify stratification biomarkers and novel interventions for autism spectrum disorder (ASD). Within the program, the Longitudinal European Autism Project (LEAP) has recruited and comprehensively phenotyped a rare sample of 76 monozygotic and dizygotic twins, discordant, or concordant for ASD plus 30 typically developing twins. The aim of this letter is to complete previous descriptions of the LEAP case-control sample, clinically characterize, and investigate the suitability of the sample for ASD twin-control analyses purposes and share some ‘lessons learnt.’ Among the twins, a diagnosis of ASD is associated with increased symptom levels of ADHD, higher rates of intellectual disability, and lower family income. For the future, we conclude that the LEAP twin cohort offers multiple options for analyses of genetic and shared and non-shared environmental factors to generate new hypotheses for the larger cohort of LEAP singletons, but particularly cross-validate and refine evidence from it.

**Electronic supplementary material:**

The online version of this article (10.1186/s13229-018-0212-x) contains supplementary material, which is available to authorized users.

## ᅟ

In Europe, 1 to 2% of the general population is diagnosed with autism spectrum disorder (ASD). ASD frequently co-occurs with other neurodevelopmental and psychiatric conditions [1, 2], persistently reduces adaptive functioning as well as quality of life, and is associated with premature mortality [3–5]. The genetic and environmental interplay driving the biological trajectories leading to ASD phenotypes remains poorly understood, and interventions are currently not targeting primary ASD etiologies. Althougth conceptualized as a neurodevelopmental disorder, the diagnosis of ASD is exclusively behavior-based, with no validated biomarkers being available to support diagnostic assessment or evaluate outcomes. Thus, a paramount objective of contemporary biomedical research on ASD is the identification of genetic, neurobiological, and other somatic markers as well as cognitive features that reliably characterize (subgroups of) autism and inform the development of novel treatment options. In order to achieve this objective, EU-AIMS (eu-aims.eu), the to date largest cross-disciplinary European network of autism researchers, was established.

EU-AIMS includes the clinical research project Longitudinal European Autism Project (LEAP) that has collected over 700 participants with ASD or typically developing (TD) controls across a multitude of European specialist ASD-centers. Protocol and sample details have been previously published in this journal [6, 7]. As a separate arm within LEAP, monozygotic (MZ) and dizygotic (DZ) twins, discordant (one twin diagnosed with ASD), or concordant (both twins diagnosed with ASD) for ASD plus TD control twins have been recruited. Using largely the common LEAP-protocol, data from the LEAP twins have been collected to enable analyses that can disentangle genetic vs. environmental factors for any putative risk marker of ASD found within the larger LEAP case-control cohort. Research using the twin design is particularly informative to study potential causes of ASD, as it provides maximum control of possible confounds, such as sex, age, socioeconomic status, family environment, and varying genetic backgrounds [8]. The purpose of this letter is to complete previous descriptions of the LEAP case-control sample and protocol [6, 7] by providing twin sample characteristics and analyzing options, using multiple levels of analysis.

The LEAP twin cohort includes *N* = 106 child, adolescent, and adult twins, 96 recruited from the Roots of Autism and ADHD Twin Study Sweden (RATSS) [9], and 10 at King’s College London (Tables 1 and 2). The LEAP twin protocol differs from the RATSS protocol, by both further deepening the cognitive/behavioral phenotyping with questionnaires, psychometric tests, eye-tracking paradigms and structural magnetic resonance imaging (sMRI) as well as magnet resonance spectroscopy (MRS) assessments (see Additional file 1: Table S1). Clinical consensus diagnosis of ASD, based on ICD-10, DSM-IV-TR, or DSM-5 criteria, is supported by results from standardized diagnostic tools, such as the Autism Diagnostic Interview–Revised (ADI-R) [10] and the Autism Diagnostic Observation Schedule-Second Edition (ADOS-2) [11, 12]. Autistic traits are measured using the parent-rated standard form of the Social Responsiveness Scale-Second Edition (SRS-2) [13, 14]. ADHD-symptoms are assessed using parent and self-ratings (> 16 years of age) on the DSM-5 ADHD rating scale. General intellectual abilities (GAI) are measured with the Wechsler Intelligence Scales for Children or Adults-IV (WISC-IV/WAIS-IV/WASI-II), and intellectual disability (ID) was defined when GAI below 70. Adaptive behavior is assessed using the parent interview version of the Vineland Adaptive Behavior Scales-Second Edition (VABS-2), as well as parent and self-ratings with the Columbia Impairment Scale (CIS). Twins without any neurodevelopmental or other disorder were defined as TD. The twin research was approved by the Ethical Review Boards in Stockholm and at Kings College. Group differences on categorical variables were calculated using chi-squared test, on rank and continuous variables using Kruskal-Wallis test, and Mann-Whitney test was applied as a post hoc test for pairwise comparisons between the groups. We used the Statistical Package for Social Sciences (IBM SPSS version 23), and a two-tailed *p* < .05 was adopted for all statistics.

**Table 1:** LEAP twin sample characteristics (*N* = 106)

	Twin-pairs concordant for ASD *n* = 10	Twin-pairs discordant for ASD *n* = 28	Twin-pairs with typical development *n* = 15
Sex % males	80.0	62.5	46.7
Age (in years)			
Mean (SD)	15.3 (3.4)	15.8 (4.4)	16.8 (2.8)
Ethnicity %	Caucasian = 100	Caucasian = 88.9 Black = 3.7% Mixed = 7.4%	Caucasian = 100
Maternal education % > high school exam	30.0	48.1	53.8
Paternal education % > high school exam	44.4	41.2	46.2
Annual household income median €	34,000–45,000	45,000–68,000	68,000–90,000

**Table 2:** LEAP twin sample diagnosis, ratings on symptoms and adaptive behavior (*N* = 106)

	ASD twins^a^ *n* = 48	Non-ASD twins^b^ *n* = 28	TD twins^c^ *n* = 30	Group comparison *p* value; effect size	Between differences (post hoc)
SRS-2^d^ mean (SD)	90.3 (27.2)	32.9 (23.9)	30.4 (25.1)	< .001; η2 = .57	a > b, c
ADI-R mean (SD)					
Total score	30.4 (15.0)	7.8 (8.1)	3.8 (4.5)	< .001; η2 = .63	a > b, c
ADOS-2^e^ mean (SD)	5.9 (2.5)	1.9 (1.1)	1.9 (1.6)	<.001; η2 = .53	a > b, c
Intellectual disability %	18.8	3.6	0	.010; φ = .29	a > c
Full-scale IQ^f^ mean (SD)	92.7 (19.6)	99.0 (14.6)	103.7 (13.0)	.051; η2 = .06	a < c
ADHD symptoms^g^ mean (SD)	22.9 (12.4)	11.3 (11.6)	4.2 (4.6)	< .001; η2 = .37	a > b, c; b > c
VABS-2^h^ mean (SD)					
Adaptive Behavior Composite	72.8 (18.4)	95.6 (17.5)	103.2 (8.9)	< .001; η2 = .41	a < b, c
CIS mean (SD)					
Self report^i^	9.7 (6.7)	10.3 (9.3)	6.3 (5.3)	.090; η2 = .05	a > c
Parent report	14.0 (8.0)	9.3 (8.7)	6.5 (6.8)	< 0.001; η2 = .17	a > b, c

Note. ^a^In ASD-discordant and concordant pairs

^b^In ASD-discordant pairs

^c^Typically developed pairs

^d^Parent-reported Social Responsiveness Scale-2

^e^ADOS-2 comparison scores

^f^WISC-IV/WAIS-IV/WASI-2

^g^DSM-5 ADHD rating scale

^h^Vineland Adaptive Behavior Scales-2

^i^Columbia Impairment Scale

The LEAP twins (38.7% female) range in age between 6 and 26 years (*M* = 16 years, SD = 3.85), with no age differences between participants with or without diagnosis. Parents with ASD twins do not differ from TD twins in terms of education, but annual household income is lower in these families (*χ*^*2*^ = 6.29; *p* = .043; *η2* = .07), particularly in families with ASD concordant twins (*U =* 86; *p* = .013; *η2* = .18). As expected, ASD twins show higher autistic trait severity on the SRS-2 (*χ*^*2*^ = 59.10; *p* < .001; *η2* = .57), and autism symptom severity on the ADI-R (total score) (*χ*^*2*^ = 63.88; *p* < .001; *η2* = .63) and ADOS (comparion scores) (*χ*^*2*^ = 53.10; *p* < .001; *η2* = .53), compared to non-ASD co-twins and TD twins. Moreover, as expected, parent-rated adaptive behavior levels on both the VABS and the CIS are lower in ASD twins compared to non-ASD co-twins and TD twins. Also, ASD twins rate themselves as more impaired on the CIS than TD twins (*U* = 394; *p* = .033; *η2* = .07), but not compared to their non-ASD co-twins. The rate of intellectual disability (ID) is 18.8% among ASD cases, 3.6% in non-ASD co-twins, and 0% in TD twins (*χ*^*2*^ = 9.13; *p* = .010; *φ* = .29). The groups differ on symptoms of ADHD (*χ*^*2*^ = 34.75; *p* < .001; *η2* = .37) and prescribed ADHD medication (central stimulants, SNRIs) (*χ*^*2*^ = 13.66; *p* = .001; *φ* = .38), with a medication rate of 40.4% in ASD twins, 19.2% in non-ASD co-twins and 0% in TD twins. If Bonferroni correction is applied, the null hypothesis is rejected if the *p* < .004.

Overall, these descriptive results indicate consistency of the twin sample with the LEAP case-control singletons in terms of SRS-2, ADOS-2, and ADI-R-score distributions for ASD cases [6]. Findings are also in line with previous ASD research regarding the impact of ASD on adaptive functioning [3, 15], and increased comorbidity with ADHD and ADHD symptoms. The mean score on the ADHD rating scale-5 [16] was around the 90th percentile in ASD cases when comparing to a normative sample. Also mirroring findings from previous research [2], other neurodevelopmental disorders and broader phenotypes were identified in several of the non-ASD co-twins. Although the proportion of ASD cases with ID appears relatively low compared to previous international research reporting an overlap of 50–70% [17], it is only slightly below more recent epidemiological data from Sweden (17.4–29.4%) [18]. Thus, the risk of an overrepresentation of ASD cases without ID in the LEAP twin sample appears limited. The inclusion of twins concordant (7 MZ and 3 DZ pairs) and discordant (10 MZ and 18 DZ pairs) for ASD, as well as TD twins (10 MZ, 5 DZ), makes the cohort suitable for twin co-twin approach disentangling and controlling for genetic and environmental factors [19]. Particularly, in including MZ twin pairs discordant for ASD, we have the opportunity to elucidate environmental risk factors and to test if any found association exists independent of genetic influences. To date, only a few of studies have applied a discordant MZ twin pair design in ASD using smaller samples than the LEAP cohort [2, 19].

Ongoing analyses on the LEAP twins include full genetic characterization of the twin pairs using a genome-wide screen of copy number variants and whole genome sequencing analysis. MRS data will be used to examine the glutamate/glutamine and GABA excitation/inhibition imbalance hypothesis [20]. We intend to investigate cognitive processes, both at the level of higher constructs and at the more basic perceptual level, that have been suggested to model ASD and its severity [21]. These analyses will extend recent twin findings on the RATSS cohort, for instance, on resting state connectivity alterations and fetal and postnatal metal dysregulation in ASD [22, 23]. In order to fully account for the statistical requirements of the twin/co-twin design (estimating the between-pair or within-pair effect), and allowing for inclusion of both categorical and dimensional autism outcomes, a statistical framework of multiple adjusted (conditional) linear regressions based on generalized estimation equations (GEE) [24] has been developed for the LEAP twins using the drgee package in R.

While the size of our LEAP twins sample is relatively small, it is to the best of our knowledge the largest collection of ASD twins that are comprehensively phenotyped and where a multitude of unique accompanying biosamples are available. The LEAP twin sample includes a significant subset (currently about 15%) of all ASD discordant twins in Sweden in the specified age range [25], enabling identification of non-shared environmental influences on the phenotype [19]. As the focus in the LEAP twin collection has been on MZ twins, the number of included DZ pairs is rather low, making heritability analyses challenging. As LEAP was a RATSS add-on, some elements from the case-control LEAP protocol had to be excluded (e.g., functional MRI) to minimize participant burden. A possible limitation in twin samples is that the finding may not be generalizable to non-twin populations. Nevertheless, as the LEAP twin cohort shares its data collection protocol with the LEAP case-control cohort, it is possible to cross-validate findings from the LEAP case-control cohort in LEAP twins, opening up the possibility to replicate singleton findings in a twin sample, controlling for genetic factors.

Lessons learnt are the complexity of identifying and recruiting twins even in case of excellent prerequisites (access to national twin register), collecting complete and usable data-sets for both twins (e.g., from sMRI, MRS) when aiming for comprehensive phenotyping, certain ASD subpopulations (e.g., “pure” ASD cases without comorbidities), and clearly phenotypically ASD discordant and concordant pairs. Given the rarity of discordant ASD twins in countries of low population, identification and recruitment must be a national, long-term, and well-funded effort. Access to nationwide population-based registry data on twins screened for neurodevelopmental disorders has been invaluable. We also recommend to conduct as many assessments online (testing, questionnaires) and offer the opportunity to participate in more demanding assessments (e.g., MRI, biosampling) in several regional sites to reduce the burden of travel and time consumption, and to take advantage of perhaps already established contacts to clinical and research units closer to home. Alternatively, families expressed the wish to perform the visits during weekends, due to school and working obligations. Another lesson learnt was the apparent beneficial effect of not only fully reimbursing the twins and their families for travel, accommodation, loss of income, and extra expenses, but also to pay a compensation (€200 for RATSS, €50 for LEAP) to each twin for participation, as well as to provide extended personal support by a research nurse taking care of all queries and practicalities. Finally, we also like to emphasize the importance of all participants getting annual updates on the twin research regarding progress, developments, and publications.

## Additional file

Additional file 1: **Table S1.** Summary of Study protocol for the LEAP twins. (DOCX 33 kb)

## Abbreviations

ADHD: Attention-deficit/hyperactivity disorder
ADI-R: Autism Diagnostic Interview-Revised
ADOS-2: Autism Diagnostic Observation Schedule Second Edition
ASD: Autism spectrum disorder
CiS: Columbia Impairment Scale
DSM-5: Diagnostic and Statistical Manual of Mental Disorders, Fifth Edition
DSM-IV-TR: Diagnostic and Statistical Manual of Mental Disorders, Fourth Edition text-revision
DZ: Dizygotic
EU-AIMS: European Autism Interventions-A Multicentre Study for Developing New Medications
GABA: Gamma-aminobutyric acid
GAI: General intellectual abilities
ICD-10: International Statistical Classification of Diseases and Related Health Problems, tenth Revision
ID: Intellectual disability
LEAP: Longitudinal European Autism Project
MRI: Magnetic resonance imaging
MRS: Magnet resonance spectroscopy
MZ: monozygotic
RATSS: Roots of Autism and ADHD Twin Study Sweden
sMRI: Structural Magnetic Resonance Imaging
SNRI: Serotonin–norepinephrine reuptake inhibitor
SRS-2: Social Responsiveness Scale, Second Edition
TD: Typically developing
VABS-2: Vineland Adaptive Behavior Scales Second Edition
WAIS-IV: Wechsler Adult Intelligence Scale-Third Edition/Fourth Edition
WASI-II: Wechsler Abbreviated Scales of Intelligence-Second Edition
WISC-IV: Wechsler Intelligence Scale for Children-Fourth Edition
