# Supplementary material for: EU-AIMS Longitudinal European Autism Project (LEAP): the autism twin cohort
Source: Mol Autism. 2018 Apr 13;9:26. doi: 10.1186/s13229-018-0212-x (PMC5899373; doi:10.1186/s13229-018-0212-x)
Supplement: Supplementary file 1 — Table S1. Summary of Study protocol for the LEAP twins. (DOCX 33 kb) [file 13229_2018_212_MOESM1_ESM.docx]

**Table S1** Summary of Study protocol for the LEAP twins

| **Domain / Task** | **Test/questionnaire** |
| --- | --- |
| Clinical ASD diagnosis | - Autism Diagnostic Interview-Revised (ADI-R)^a,c^ |
|  | - Autism Diagnostic Observation Schedule (ADOS or ADOS-2)^a,c^ |
|  |  |
| Dimensional measures of ASD symptoms | - Social Responsiveness Scale-2^nd^ Edition (SRS-II)^a,p^ |
|  | - Repetitive Behaviour Scale-Revised (RBS-R)^p^ |
|  | - Sensory Profile (SP)^a,s,p^ |
|  | - Children’s Social Behaviour Questionnaire (CSBQ)^p^ - Adults’ Social Behaviour Questionnaire (ASBQ)^p,s^ |
|  | - Autism Quotient (AQ), AQ-Adol, AQ-Child^a,p,s^ |
|  |  |
| Comorbidities | - DSM-5 ADHD rating scale^p,s^ |
|  | - Kiddie Schedule for Affective Disorders and Schizophrenia (K-SADS)^a,c^ - The Structured Clinical Interview for DSM-IV (SCID, axis I)^a,c^ |
|  | - The Diagnostic Interview for ADHD in adults (DIVA)^a,c^ |
|  |  |
| Quality of life / Adaptive Behaviour | - Vineland Adaptive Behaviour Scale-2^nd^ Ed (VABS-2)^a,p^ |
|  | - Columbia Impairment Scale (CIS)^p,s^ |
|  |  |
| Medical or Psychiatric History | - NIH ACE Subject Medical History Questionnaire^p,s^ |
|  | - NIH ACE Family History Form^p,s^ |
|  |  |
|  |  |
| Cognitive and Psychological Profile | - Wechsler Intelligence Scales for Children or Adults-IV (WISC-IV/WAIS-IV/WASI-II)^a,c^ |
|  | - Leiter-revised scales in combination with the Peabody Picture Vocabulary Test Third Edition^a,c^ |
|  | - Probabilistic reversal learning^c^ |
|  | - Un/Segmented block design task^c^ |
|  | - Animated shapes narratives task^c^ |
|  | - Reading the Mind in the Eyes task (RMET)^a,c^ |
|  | - Sandbox continuous false belief task^c^ |
|  |  |
| Neuroimaging | - Magnet Resonance Spectroscopy ^c^ - Magnetic Resonance Imaging^c^ |
|  |  |
| Eye-tracking | - Natural scenes: static and dynamic^c^ |
|  | - Gap overlap^c^ |
|  | - Implicit false belief ^c^ |
|  | - Pupillary light reflex^c^ |
|  | - Biological motion^c^ |
|  | - Emotion matching task^c^ |
|  | - Change detection task^c^ |
|  |  |
| Biological samples | - Blood sample (for genomic analyses)^a,c^ |
|  | - Saliva (for genomic analyses where blood samples cannot be obtained and for epigenetics)^a,c^ |
|  | - Urine (at home, for biochemical biomarkers)^a,c^ |
|  |  |
| Assessment of clinical symptoms in both biological parents | - Social Responsiveness Scale (SRS-2)^s^ - DSM-5 ADHD rating scale^s^ |

*Note*. Number of tests used may vary depending on participants age and presence of an intellectual disability (see Loth et al., 2017). ^a^Included in the RATSS study; c, clinician-rated/administrated; p, reported by parent; s, self-reported. ADHD, attention-deficit hyperactivity disorder; ASD, autism spectrum disorder; Base, baseline assessment wave; DSM, Diagnostic and Statistical Manual of Mental Disorders; NIH ACE, US National Institutes of Health Autism Centers of Excellence.
